# Iris melanocytic tumours in New Zealand/Aotearoa: presentation, management and outcome in a high UV exposure environment

**DOI:** 10.1038/s41433-022-02017-2

**Published:** 2022-03-25

**Authors:** Micah E. J. Rapata, Jie Zhang, William J. Cunningham, Peter W. Hadden, Dipika V. Patel, Charles N. J. McGhee

**Affiliations:** 1 Department of Ophthalmology, New Zealand National Eye Centre, Faculty of Medical and Health Sciences, University of Auckland, Auckland, New Zealand; 2 Department of Ophthalmology, Greenlane Clinical Centre, Auckland District Health Board, Auckland, New Zealand; 3 Eye Institute, 123 Remuera Road, Auckland, New Zealand

**Keywords:** Uveal diseases, Outcomes research

## Abstract

**Background/Objectives:**

Iris melanoma, a rare intraocular malignancy, represents the smallest subgroup of uveal melanoma. This first, comprehensive study of iris melanocytic lesions in the high ultraviolet environment in New Zealand/ Aotearoa (NZ) examines diagnosis, management and outcomes.

**Subjects/Methods:**

Retrospective study of iris melanocytic tumours referred to tertiary referral centres in Auckland, NZ, over 20 years (1999-2018). Data analysed include demographics, tumour characteristics, histology, genetic analyses, treatment modalities, recurrence, metastasis, 5-year and overall survival.

**Results:**

Cohort (N = 51) was predominantly NZ European (98.0%) with no indigenous Māori, or Pasifika. Median age at presentation was 58 years. Tumours involved a median of two clock hours of iris. The posterior tumour margin extended to the anterior chamber angle in 22 patients (45.8%). Management included initial observation 54.9%, iridectomy/excision biopsy 29.4%, irido-cyclectomy 7.8%, plaque radiotherapy 7.8%, proton beam radiotherapy 7.8%, and ultimately enucleation 17.6%. Histology was performed in 19 cases (37%) with 16 confirmed melanomas (84%). Mean follow-up 4.2 years with median visual acuity of 6/7.5 two years post intervention. Melanoma-related metastasis and mortality occurred in two cases with five-year melanoma-related mortality of 2.0%.

**Conclusion:**

In a climate with high ultraviolet exposure iris melanocytic tumours occurred almost exclusively in NZ Europeans, however, the majority of cases were category T1, possibly reflecting early diagnosis in the NZ health system. Nonetheless, >50% underwent surgery or radiotherapy, often utilising more than one modality. A high index of suspicion and early referral of iris melanocytic lesions should be considered in regions with high UV exposure.

## Introduction

Iris melanoma is a rare intraocular malignancy representing the smallest subgroup of the uveal melanomas [1]. Iris melanomas are estimated to account for only 4% of uveal melanomas, with the remaining originating in the choroid (90%) and in the ciliary body (6%) [2]. Worldwide, only approximately 7000 new cases of uveal melanoma are diagnosed each year, most occurring in white Caucasian populations of Europe and North America with only a few hundred of these cases being iris melanoma [3]. Most iris melanomas occur in individuals of Caucasian and European descent [3]. Although an association has been long-established between ultraviolet light (UV) exposure and cutaneous melanoma, its association with uveal melanoma is less established [4].

Due to the low global incidence of iris melanoma, to date, the majority of large studies of iris melanoma have been conducted in the United States and Europe. The only prior study of iris melanoma in New Zealand/Aotearoa (NZ) was conducted by Michaloval et al in 2001, however, this study included only 16 cases [5]. NZ is a nation in the South Pacific (population 5.1 million, 2020), that comprises two main islands (North and South) and provides government-funded universal health care services for all citizens (very similar to but preceding the British National Health System) [6, 7]. Due to the relative paucity of published data on iris melanoma a larger NZ study of iris melanoma may be useful in providing contemporary information of both global and national interest.

Unfortunately, New Zealand and Australia have the highest incidence of cutaneous melanoma internationally and NZ has the highest associated mortality, with a much greater environmental UV exposure than countries of comparable latitude in the Northern hemisphere [8, 9]. However, data on iris melanoma per se is limited, therefore the aim of this study was to identify iris melanocytic tumours (i.e., iris melanomas and/or iris tumours with a high index of suspicion for melanoma), managed in two tertiary centres in Auckland, NZ in order to determine the demographics, diagnostic features, management and outcomes. New Zealand has a government-funded, public hospital service, with almost 150 consultant ophthalmologists as well as >600 community-based optometrists serving the population of 5.1 million over a geographic area slightly larger than the United Kingdom (UK). Therefore, access to high quality eye care and early identification of eye disease was anticipated.

## Subjects and methods

A retrospective analysis was performed of patients with a clinical diagnosis of iris melanocytic tumours managed at Greenlane Clinical Centre, Auckland District Health Board, NZ; and Eye Institute, Auckland, NZ in the 20-year period January 1, 1999 to December 1, 2018. Both facilities are large tertiary referral centres in the North Island of NZ where the majority of iris melanocytic tumours from the North Island (population 3.7 million, 77% of population) are referred. Two patients were also included from Dunedin (South Island) as they had been referred to a Greenlane Clinic specialist (PWH) with regard to their iris melanocytic tumours.

This study was approved by the Auckland District Health Board research committee. All patients were examined by fellowship trained ophthalmologists (CMcG, and PWH). Inclusion criteria were a clinical and/or histological diagnosis of iris melanoma or naevus suspicious of being melanoma. Data were collected regarding demographics, tumour features, associated features, outcomes at five years, histological tests, genetic tests and management modalities. Data were identified from an established prospective database and review of clinical notes, clinical photographs, and histopathological reports.

Demographic data included patient age at presentation, gender, self-reported ethnicity, iris colour (blue, brown, green) and affected eye. Examination features recorded at presentation included: best corrected visual acuity (BCVA) of affected eye, intraocular pressure (IOP), and status of anterior chamber (normal, hyphaema, cells/flare).

Tumour features included tumour shape (round, oblong, geographic, diffuse/flat involving the entire iris), tumour surface (irregular, smooth), tumour configuration (nodule, flat), tumour colour (melanotic, amelanotic, mixed), quadrantic tumour epicentre (superior, inferior, temporal, diffuse), antero-posterior tumour epicentre (pupillary margin, midzone, iris root, anterior angle of chamber, diffuse), anterior tumour margin (pupillary margin, midzone, iris root, angle of anterior chamber), posterior tumour margin (pupillary margin, midzone, iris root, angle of anterior chamber, ciliary body), and tumour category (T1, T2, T3, and T4) [3]. Associated tumour features included ectropion uveae, corectopia, dilated feeder episcleral vessels, dilated feeder iris vessels, intrinsic vascularity, cataract, ocular melanocytosis, extraocular extension, and secondary glaucoma.

Tumour samples were obtained by local resection or enucleation. For iris tumours that were biopsied or excised, histological results were recorded. Iris lesions were then classified as benign naevi or malignant melanoma. Malignant melanoma were classified by cell type (spindle, epithelioid, mixed, or unknown). Genetic analysis was performed in a minority of cases but all genetic analysis outcomes were recorded (monosomy 3, no genetic changes, other changes, failed test).

Management was classified into six groups: (1) observation with photography: (2) iridectomy, (3) irido-cyclectomy, (4) plaque radiotherapy, (5) proton beam radiotherapy, and (6) enucleation (primary, secondary). For proton beam radiotherapy, patients were referred to the Liverpool Ocular Oncology Service, United Kingdom. Some patients underwent multiple treatment modalities. The type and number of treatments undertaken were also recorded. The length of follow-up was measured by calculating the length of time between initial presentation and the most recent clinical appointment. The best corrected visual acuity was also recorded approximately two years after the primary treatment was undertaken. Outcomes within five years included local tumour recurrence, lymph node metastasis, melanoma-related metastasis, and melanoma-related death (notably as many patients returned to their referring centre for longer term follow-up but current health status was ascertained for all subjects). Iris-melanoma metastasis related and unrelated mortality in the 20 year study period was recorded.

## Results

During the 20-year study period, a total of 51 patients with a presumptive clinical or histological diagnosis of iris melanoma were identified.

### Demographics

The median patient age at presentation was 58.0 years (*n* = 51). The cohort was predominantly NZ European, (*n* = 50, 98.0%) with one patient of Chinese descent (2.0%). No patient was indigenous to NZ (Māori) or the Pacific Islands (Pacific peoples). The most common iris colour was blue (*n* = 38, 84.4%). The right eye was affected in 28 patients (54.9%) and there were no bilateral cases. The median BCVA of the affected eye at presentation was 6/7.5 (range 6/4.4 to hand movements). The median IOP at presentation was 16 mmHg (range 10 to 39) **(**Table 1**)**.

**Table 1:** Demographic data and ocular features on presentation of patients with iris melanocytic tumours (*N* = 51). For each category, the number of patients with available information was noted as “n”. BCVA = best-corrected visual acuity; IOP = intra-ocular pressure; and HM = hand movements.

Age at presentation (*n* = 51)		Gender (*n* = 51)	
Median (range)	58.0 years (15.0–89.8 years)	Male	23 (45.1%)
Mean	55.4 years	Female	28 (54.9%)
**Self-reported ethnicity (*n*** = **51)**		**Iris colour (*n*** = **45)**	
NZ European	50 (98.0%)	Blue	38 (84.4%)
Chinese	1 (2.0%)	Brown	3 (6.7%)
		Green	4 (8.9%)
**Affected eye (*n*** = **51)**		**BCVA of affected eye (*n*** = **48)**	
Left	23 (45.1%)	Median (range)	6/7.5 (6/4.4–HM)
Right	28 (54.9%)		
**Status of anterior chamber (*n*** = **51)**		**IOP of affected eye (*n*** = **40)**	
Normal	47 (92.2%)	Median (range)	16 mmHg (10 – 39 mmHg)
Hyphaema	2 (3.9%)		
Cells/flare	2 (3.9%)	Mean	18 mmHg

### Tumour features

**Fig. 1:**
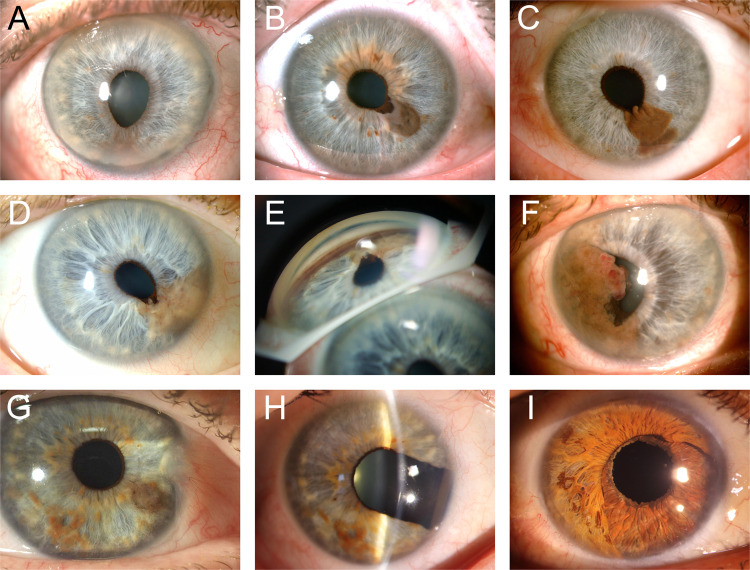
Malignant melanotic iris tumours display a variety of tumour features. **A** a discrete malignant iris melanoma with corectopia subsequently associated with severe glaucoma; **B** a small malignant spindle B iris melanoma that was completely excised; **C** an elevated malignant iris melanoma with recent growth extending from pupil margin towards the angle, subsequently completely excised; **D** a 3-clock hour diffuse coffee coloured mixed spindle B and epithelioid cell iris melanoma; **E** angle appearance of case **D**, showing extension toward the angle with widespread pigment dispersion – subsequent excision provided recurrence-free control at 14 years; **F** a large, vascular, low grade malignant melanoma arising in a longstanding naevus; **G** a small malignant iris melanoma at presentation; **H** the same case eight years post-iridectomy with 6/6 visual acuity and no tumour recurrence; **I** five-clock hour iridectomy repair after excision and proton beam therapy for malignant melanoma, by combined phacoemulsification, IOL and endocapsular artificial iris with 6/6 visual acuity at 5 years.

Tumour features and category are summarized in detail in Table 2. Images of some tumours are presented in Fig. 1. The median number of clock hours involved by iris melanocytic tumours was two, 23 tumours (50.0%) had no distinctive shape and were classified as ‘geographic’. The majority of tumours were pigmented and classified as melanotic (*n* = 37, 77.1%); the anterior tumour margin was at the pupillary margin in 25 patients (54.3%); and 47 tumours (95.9%) were classified as stage T1, where the tumour was limited to the iris [3]. Ten tumours (20.4%) were T1c, where there was secondary glaucoma. No cases were T2c (tumour confluent with or extending into the ciliary body, choroid, or both, with secondary glaucoma), T3 (T2 with scleral extension), or T4 (with extra-scleral extension) [3].

**Table 2:** Descriptive features, category, and associated features present at time of diagnosis and prior to any intervention of iris melanocytic tumours included in this study. Note: 26 eyes exhibited more than one feature. For each category, the number of patients with clinical information available was noted as “n”.

Clock hours involved (*n* = 48)		Anterior tumour margin (*n* = 46)	
Median (range)	2.0 (0.25–10.0)	Pupillary margin	25 (54.3%)
Mean	2.2	Midzone	14 (30.4%)
**Tumour shape (*n*** = **46)**		Iris root	5 (10.9%)
Geographic	23 (50.0%)	Ant. chamber angle	2 (4.3%)
Round	14 (30.4%)	**Posterior tumour margin (*n*** = **48)**	
Diffuse/flat	5 (10.9%)	Pupillary margin	1 (2.1%)
Oblong	4 (8.7%)	Midzone	5 (10.4%)
**Tumour surface (*n*** = **41)**		Iris root	18 (37.5%)
Irregular	23 (56.1%)	Ant. chamber angle	22 (45.8%)
Smooth	18 (43.9%)	Ciliary body	2 (4.2%)
**Tumour configuration (*n*** = **46)**		Tumour category* (*n* = **49)**	
Nodular	23 (50.0%)	T1	47 (95.9%)
Flat	23 (50.0%)	* T1a*	31 (63.3%)
**Tumour colour (*n*** = **48)**		* T1b*	6 (12.2%)
Melanotic	37 (77.1%)	* T1c*	10 (20.4%)
Amelanotic	5 (10.4%)	T2	2 (4.1%)
Mixed	6 (12.5%)	* T2a*	1 (2.0%)
**Quadrantic tumour epicentre (*n*** = **49)**		* T2b*	1 (2.0%)
Infero-temporal	21 (42.9%)	T2c, T3, or T4	0
Infero-nasal	14 (28.6%)	**Associated features (*n*** = **51)**	
Supero-nasal	5 (10.2%)	Ectropion uveae	21 (41.2%)
Diffuse	5 (10.2%)	Corectopia	20 (39.2%)
Supero-temporal	4 (8.2%)	Cataract	19 (37.3%)
**Antero-posterior tumour epicentre (n** = **46)**		Secondary glaucoma	12 (23.5%)
Pupillary margin	1 (2.2%)	Dilated feeder episcleral vessels	5 (9.8%)
Mid-zone	27 (58.7%)	Intrinsic vascularity	3 (5.9%)
Iris root	13 (28.3%)	Pseudophakia	3 (5.9%)
Ant. Chamber angle	2 (4.3%)	Dilated feeder iris vessels	2 (3.9%)
Diffuse	3 (6.5%)	Ocular melanocytosis	2 (3.9%)
		Extraocular extension	0 (0.0%)

^*^Tumour categories: T1 = tumour limited to iris [T1a: <3 clock hours, T1b: >3 clock hours, T1c: with secondary glaucoma]. T2 = tumour confluent with or extending into ciliary body, choroid or both [T2a: tumour confluent with or extending into the ciliary body], without secondary glaucoma, T2b: tumour confluent with or extending into the ciliary body and choroid, without secondary glaucoma, T2c: tumour confluent with or extending into the ciliary body, choroid, or both, with secondary glaucoma. T3 = T2 + scleral extension. T4 = tumour with extra-scleral extension [3].

### Tumour associated features

The most frequency observed tumour associated features were ectropion uveae (41.2%), corectopia (39.2%), cataract (37.3%), and secondary glaucoma (23.5%).

### Histopathology and Cytogenetics

A minority of patients (*n* = 19, 37.3%) had histology performed on tumour biopsy-excisions (*n* = 16, 31.4%) or enucleations (*n* = 4, 7.8%). One patient underwent both tumour excision biopsy and later enucleation. Of the 19 melanocytic tumours, three were reported as benign. All four enucleations were histologically confirmed to be iris melanomas. Of the 16 histologically-confirmed melanomas 7 (43.8%) were reported as mixed spindle and epithelioid cells, and 6 (37,5%) were predominantly spindle cells (Table 3). Eleven tumour samples underwent fluorescence in-situ hybridization (FISH) genetic analysis but due to inadequate tissue samples only nine samples were reported; four (44.4%) displayed monosomy three and another three (33.3%) had other genetic changes (Table 3).

**Table 3:** The histopathology results of eyes that underwent biopsy or enucleation (*n* = 19). Histological sub-classifications and genetic testing results are provided where available. The predominant technique for detecting genetic abnormalities in biopsied tissue was fluorescence in-situ hybridization (FISH).

Histopathology (*n* = 19)	
Confirmed melanoma (*n* = 16)	
Spindle	6 (37.5%)
Epithelioid	1 (6.3%)
Mixed	7 (43.8%)
Unknown	2 (12.5%)
Confirmed benign tumour (*n* = 3)	
**Genetic analysis (*n*** = **9)**	
Monosomy 3	4 (44.4%)
No genetic abnormality	2 (22.2%)
Other genetic abnormalities including	3 (33.3%)
* RREB1 (6p25), D6Z1 (6cen) - Gain of chromosome 6p*	1 (11.1%)
* MYB (6q23) - Loss of chromosome 6q*	
* MYC (8q24), D8Z2 (8cen) - Gain of chromosome 8*	
* VHL (3p25), D3Z1 (3cen) - No loss but gain of chromosome 3*	
* Gain of CEP 8 and MYC gene loci*	1 (11.1%)
* Loss of 1p36, 3q27, and 9p21*	1 (11.1%)

### Clinical management

Twenty-eight patients (54.9%) were initially observed with serial photography, and eight subsequently required a secondary treatment modality **(**Table 4**)**. Fifteen patients (29.4%) underwent iridectomy with 10 requiring another form of treatment. Irido-cyclectomy was performed in four patients (7.8%), none required a secondary treatment. Plaque radiotherapy was performed in four patients (7.8%), three of which requiring a secondary treatment. Proton beam radiotherapy was performed in four patients (7.8%), one of which had subsequent enucleation. Overall, nine eyes were enucleated (17.6%); five (7.8%) as the primary treatment (two with glaucoma) and four (7.8%) secondary enucleations due to tumour growth with or without intractable glaucoma (one case was initially observed with photography and was the only patient to undergo a secondary enucleation without glaucoma) **(**Table 4**)**.

**Table 4:** Treatment modalities in patients (*N* = 51) with iris melanocytic tumours. Some patients required more than one method of treatment. The length of follow-up was defined as the time from presentation until the last recorded clinic appointment. Outcomes noted at “5 years” were those that occurred within a five-year period from the time of presentation. Best corrected visual acuity (BCVA) was measured at two years post primary intervention.

Treatment (*n* = 51)			
Observation: photography	28 (54.9%)	Proton-beam radiotherapy	4 (7.8%)
Iridectomy	15 (29.4%)	Enucleation	9 (17.6%)
Irido-cyclectomy	4 (7.8%)	* Primary*	*5 (9.8%)*
Plaque radiotherapy	4 (7.8%)	* Secondary*	*4 (7.8%)*
**Patients who underwent** > **1 method of treatment (*n*** = **51)**			
Observation: photography	8 (15.7%)	Plaque radiotherapy	3 (5.9%)
Iridectomy	10 (19.6%)	Proton beam radiotherapy	1 (2.0%)
Irido-cyclectomy	0 (0.0%)		
**Length of follow-up (*n*** = **46)**			
Median (range)		2.4 years (range 0–17.5 years)	
Mean		4.2 years	
**BCVA at 2 years post primary intervention of retained eye (*n*** = **23)**			
Median (range)		6/7.5 (6/4.8–6/60)	
**Outcomes at 5 years (*n*** = **51)**			
Local tumour recurrence		2 (3.9%)	
Lymph node metastasis		2 (3.9%)	
Melanoma-related systemic metastasis		1 (2.0%)	
Melanoma-related death		1 (2.0%)	
**Mortality in 20 year study period (*n*** = **51)**			
Overall mortality		6 (11.8%)	
* Iris melanoma metastasis related*		2 (3.9%)	
* Non-iris melanoma related*		4 (7.8%)	

The longest follow-up period recorded was 17.5 years, with mean follow-up of four years two months and median follow-up of two years five months. The median BCVA at two years post-intervention was 6/7.5 (*n* = 23, range 6/4.8 to 6/60). Outcomes at five years include local tumour recurrence in 2 patients (3.9%), lymph node metastasis in 2 (3.9%), melanoma-related distant metastasis in 1 (2.0%) and melanoma-related death in 1 patient (2.0%). During the 20-year study period six patients (11.8%) died of causes including: iris melanoma metastasis (*n* = 2, 3.9%), cutaneous melanoma metastatic (*n* = 2, 3.9%), bowel perforation (*n* = 1, 2.0%) and acute renal failure (*n* = 1, 2.0%).

## Discussion

In the 20 year study period, 51 cases of iris melanocytic tumours were managed at two large tertiary referral centres that serve the North Island of New Zealand / Aotearoa. In this high UV radiation environment, the cohort was predominantly NZ European with blue irides. The most common tumour features included: a median of 2 clock hours of iris involvement, geographic shape, irregular surface, inferior iris location, tumour at pupil margin, and extension to the anterior chamber angle. Ectropion uveae, corectopia, cataract, and secondary glaucoma were the most common associated features. However, the vast majority (96%) of tumours were category T1 limited to the iris with no scleral extension. Where histology was performed the majority of melanocytic lesions were confirmed as malignant. Initial management included regular observation (54.9%) but ultimately more than a third (37.2%) underwent excisional iridectomy/biopsy (29.4%) or Irido-cyclectomy (7.8%), nine (17.6%) underwent enucleation and other modalities were plaque radiotherapy (7.8%) or proton-beam radiotherapy (7.8%). The median visual acuity before and 2 years after treatment was unchanged at 6/7.5 (excluding eyes undergoing enucleation). Outcomes at 5 years were generally favourable with 3.9% local tumour recurrence and lymph node metastasis and only one (2.0%) melanoma-related metastasis and death. Over the 20 years only 2 subjects (3,9%) died of iris melanoma related metastatic disease but notably 2 other subjects died of metastatic cutaneous melanoma, another high UV radiation exposure related disease in susceptible individuals.

Interestingly, iris melanocytic tumours were not observed in any individuals with darkly pigmented skin or irides, including Māori and Pacific peoples. Brown irides were noted in only 6.7% of the study population, lower than the 8.6% with brown irides in a multi-national, multi-centre study of 131 biopsy-proven iris melanomas across Europe and USA [10]. One case of iris melanoma was clinically diagnosed in a woman of Chinese descent. New Zealand/Aotearoa has a predominantly European Caucasian population (70.2%), with large minorities of indigenous Māori (16.5%), Pacific peoples (8.1%), and Asian (15.1%) populations [11]. Uveal melanoma is extremely rare in individuals with darkly pigmented skin and irides [12, 13]. A previous, smaller, NZ study also reported no cases in those of Māori descent [5]. In a major United States study of uveal melanoma (*N* = 8022), 98% were Caucasian, 1% Hispanic, and <1% were Asian, African-American and Native American [2, 13]. There are no previously published cases of iris melanoma in Māori or Pasifika and only a single case of choroidal melanoma in a person of Māori descent [12].

Iris tumours involved the inferior quadrants of the iris in 71.5% of eyes, similar to the rate reported in neighbouring Australia (78.4%) [3, 14], Europe and USA (79.2%) [10]. This may reflect the geometry of the eye and surrounding structures that limit the exposure of anterior ocular sites to direct solar irradiance [15, 16]. Conway et al., in a similar-sized 20-year study, in Sydney, Australia, observed a higher mean number of clock hours of iris involvement compared to the current study (4.3 vs. 2.2), greater irido-corneal angle involvement (64.7% vs. 45.8%), and tumour nodularity (78.4% vs. 50.0%) [14]. As expected, biopsy-proven iris melanomas may have a higher mean number of clock hours of iris involved (2.5) than the current study because larger lesions are more likely to be biopsied [10]. In contrast, a large USA based study of iris melanoma (*N* = 432) showed lower proportions of ectropion uveae (28% vs 41.2%), corectopia (26% vs 39.2%), and cataract (14% vs 37.3%), but demonstrated higher rates of hyphaema (9% vs. 3.9%) and extraocular extension (5% vs. 0%) than the current study [3]. A multi-centre study of biopsy-proven iris melanomas (*N* = 131) had similar rates of ectropion uveae (42.6% vs. 41.2%), hyphaema (3.1% vs. 3.9%) and glaucoma (26.8% vs. 23.5) but lower rates of corectopia (25.9% vs. 39.2%) and cataract (17.5% vs. 37.3%) compared to the current study [10]. These differences in presentation may be due to limitations inherent to our relatively small cohort sizes in Australasia and differences in study population, but could point to genuine differences in our populations or environmental exposure to solar irradiation. Although there is no published evidence of a link between advanced clinical features such as ectropion uveae or corectopia and UV exposure, recent research has demonstrated that chromosome 3 and 8q aberrations in uveal melanoma may impact on survival in patients with light iris versus dark iris colour. These results suggest a novel, possibly synergistic, effect between chromosomal abnormality and iris colour on oncogenic behaviour [17]. Notably, the majority of our cases had pale blue (84.4%) or green irises (8.9%) and we noted that some of the larger tumours were amelanotic or relatively low in pigmentation and may have arisen from prior naevi, therefore, these may have been more clinically advanced before being detected. Interestingly, a 2022 study of iris melanoma topography noted, in a predominantly blue-eyed Norwegian population, that 52% demonstrated ectropion uvea or corectopia, 55% had arisen from presumed naevi, 54% exhibited a tumour size of ≥5 mm and yet the majority were spindle cell tumours. Ultimately, widespread iris melanoma metastases occurred in only 1 of 28 cases at a mean 12 years follow-up [16].

Histology is invaluable for differentiating iris naevi from melanoma and for prognosis when used with other clinical factors [18–20]. However, due to rarity, iris melanoma prognostic data have largely been generated collectively from uveal melanoma studies limiting histological prognostication [2, 13]. Iris melanoma metastases rates may vary depending on tumour cell type; mixed cell tumours have the highest metastatic rate (10.5%) compared to epithelioid cell (6.9%) and spindle cell (2.6%) tumours [21]. However, epithelioid cytology is generally associated with a poorer prognosis in uveal melanomas [1, 18, 19]. Of the 19 patients with histological analysis, the majority (*n* = 16) were reported as malignant melanoma which may reflect that tumours with the highest degree of clinical suspicion were more likely to undergo biopsy. Conway *et al*. revealed lower proportions of epithelioid cell (2.2% vs. 6.3%) and mixed cell tumours (28.3% vs. 43.8%) but a higher percentage of spindle cell tumours (69.6% vs. 37.5%) compared to our study [14]. This differing profile may be due to the higher proportion of tumours (90% vs 37%) that underwent histology in the Australian study [14] due to a lower threshold for biopsy in Australia, or a larger number of tumours with a high degree of clinical suspicion. Biopsy-proven iris melanomas, in a multi-centre study were reported as: 5% epithelioid cell tumours and 54% spindle cell tumours, both values being in-between Australian and current New Zealand study values, however, the proportion of mixed cell tumours (28%) was very similar to the Australian study [10]. Nonetheless, allowing for the relatively short mean follow-up time (4.2 years) the prognosis of our cohort with a high percentage of mixed cell and epithelioid cell types appears reasonable.

Cytogenetic testing may similarly assist prognostication. Monosomy three was the main abnormality tested for by FISH and was identified in 44.4% of tumours that were successfully analysed. Monosomy three, seen in 50% of uveal melanomas, is the most frequently encountered genetic abnormality in uveal melanoma, and correlates strongly with metastatic disease [22]. A small series of 17 cases of iris melanoma reported monosomy three in 29% of patients [23]. FISH is the primary cytogenetic test available in NZ and a recent review recommended gene-expression profiling as the preferred prognostic test in posterior uveal melanoma as it has been validated in a multi-centre clinical trial [24].

Overall, six patients (11.8%) died during the study period but only two of these cases (3.9%) died from iris-melanoma related metastases. All seven individuals with confirmed genetic abnormalities and all 16 cases with confirmed histopathology survived at five years with the only iris-melanoma-related death at five years having neither histopathology or genetic analysis. Of those with genetic abnormalities, three died: one case (monosomy 3) died at 14 years from a cause unrelated to iris-melanoma; one case with other genetic abnormalities (row 14, Table 3) died at six years from cutaneous melanoma-related metastasis (not iris-melanoma-related); the third case, with a large number of genetic abnormalities (row 12, Table 3), died at 11 years due to metastasis of iris melanoma. Of 16 cases with confirmed histopathology, three also died: two cases with mixed spindle and epithelioid cell tumours died from causes unrelated to iris melanoma, whereas one with a predominantly spindle cell tumour died of iris melanoma metastases at 11 years.

Management of iris melanocytic tumour is highly individualized and depends on clinical features including tumour size, tumour extension or seeding into the angle, and melanoma-related glaucoma [25]. Since iris melanomas have relatively low metastasis rates (1.4%, 5.4%, and 9.8% at 3, 5, and 10 years, respectively) [3] and typically good systemic prognoses, regular observation of melanocytic lesions for growth or change in morphology is the common initial management option in the current (54.9%) and many published studies [14, 26, 27].

Plaque radiotherapy has surpassed local resection by iridectomy and enucleation in recent years as the primary treatment modality for iris melanoma [3, 18]. Conway et al. reported 43.1% of patients underwent local resection in a Sydney cohort (1980–2000) in a period wherein plaque radiotherapy was not widely available [14]. In the current study the most common interventional management approach was iridectomy (29.4%), due to the relatively well-circumscribed tumours amenable to surgery using the minimal iris touch excision (MITE) technique [28]. This technique allows complete resection of small to medium iris lesions via small corneal incisions, with minimal tumour disturbance [28]. The relatively low usage of plaque radiotherapy, and proton beam radiotherapy was due to these smaller, well circumscribed tumours, more suitable for surgical excision. Nonetheless, in half of the plaque radiotherapy cases, iridectomy was initially performed to confirm tissue diagnosis and de-bulk the tumour.

Enucleation is typically reserved for extensive tumours demonstrating seeding, often with advanced, uncontrollable secondary glaucoma [19]. In the 20 year study period a total of nine eyes (17.6%) were enucleated, five as the primary treatment (9.8%) and four as secondary enucleations (7.8%) following another initial treatment modality (one each: observation, proton beam radiotherapy, iridectomy, plaque radiotherapy). In comparison, Shields et al. in a cohort of 432 patients demonstrated secondary enucleation rates of 7.1%, 11.7%, and 18.6% at 3, 5, and 10 years respectively, while Conway et al. reported that only 2 of 51 patients (3.9%) underwent secondary enucleation with mean follow-up of 8.7 years [3, 10]. Enucleations for choroidal melanoma have typically decreased since the Collaborative Melanoma Study, which demonstrated no survival benefit over I^125^ plaque radiotherapy in medium-sized uveal tumours [14, 21]. Our sample size and mean follow-up of 4.2 years (range 0–17.5 years) may account in part for our five-year iris melanoma-related metastasis rate (2.0%) being slightly lower than that reported by Shields et al. (5.2%, *N* = 317) [22]. A much higher five-year iris melanoma-related metastasis rate in biopsy-proven melanomas (10.7%) can be explained by a higher proportion of T2-4 tumours (37% vs. 4.1%), higher rates of episcleral vessels (16.8% vs. 9.8%), dilated feeder iris vessels (7.3% vs. 3.9%), and much higher rates of intrinsic vascularity (56.5% vs. 5.9%) compared to the current study [10].

Trials of proton beam radiotherapy have demonstrated good local tumour control, eye preservation, and disease-free progression in choroidal and ciliary body melanoma [14]. Proton beam radiotherapy was used in only four patients (7.8%), partly because there is no proton beam radiotherapy centre in Australasia but suitable patients are offered treatment overseas (UK). The first proton beam radiotherapy unit in the southern hemisphere will shortly be built in Adelaide, Australia [23].

Notably the vast majority of iris melanocytic lesions in this study occurred in white Caucasians with pale irides and no indigenous Māori were affected. The association of pale skin, pale iris colour and iris melanoma has been known for some time and an aetiological relationship between light iris colour and exposure to UV radiation has been proposed [29], this is a potentially significant aetiological risk due to high solar irradiation in New Zealand and Australia. Shields et. al. succinctly summarised susceptibility factors that include: fair skin, light eye colour, inability to tan, ocular or oculodermal melanocytosis, cutaneous or iris or choroidal nevus, and BRCA1-associated protein 1 mutation [30]. However, despite global increase in cutaneous melanoma in fair-skinned individuals related to ultraviolet light exposure, the burden of uveal melanoma has been reported to be more variable: being relatively constant in England (latitude 52°N) at approximately 1/100,000 between 1979–2010; [31] increasing in both Canada (56°N) between 1992–2010 [32] and Norway (60°N) between 1993–2004; [33] and decreasing in Sweden (60°N) between 1960–1998 [33]. Notably, the two provinces with the highest incidence of uveal melanoma in Canada were at lower latitudes [32]. Closer to home, a study from Queensland, Australian (22°S) failed to identify cumulative lifetime ocular UV-B exposure as a risk factor for ocular melanoma [34]. Therefore, with the limited currently available data, it is difficult to firmly conclude whether the incidence of uveal melanoma has been increasing over the fifty years. Available data suggest there has not been a significant increase in the incidence of choroidal, ciliary body or iris melanomas in New Zealand (40°S) in the study period and two of the authors have reviewed the majority of iris, choroidal and ciliary body melanomas in New Zealand for two decades, however, anecdotally, they note a slight increase in iris melanoma referrals in the period 2018–2022. Clearly, more studies are required to confirm trends in the incidence of uveal / iris melanoma, taking into account latitude and cumulative UV exposure.

Recently it has been noted that iris and conjunctival melanoma, unlike more posteriorly located uveal melanoma, often carry a higher tumour mutational burden (TMB) and exhibit specific mutations linked with UV exposure [35]. Whole genome sequencing of iris, ciliary body and choroidal melanoma highlights that although these tumours typically have a low TMB, two subgroups have a high TMB, importantly, one group exhibits a mutation driven by UV-exposure, restricted to iris melanomas [36]. The distribution of *GNAQ* and *GNA11* mutation signatures also provides further supporting molecular evidence for a light or UV exposure dependent mechanism in uveal melanoma [37]. Recently, Goh et al (2020) noted in a review of mutations in >1000 uveal melanoma and >12,500 cutaneous melanoma samples that the aetiology of a substantial minority of uveal melanomas may be more UV-dependent than previously accepted [4].

Ultimately approximately 50% of subjects with uveal melanoma will die of metastatic disease [36], although the limited data highlights better prognosis for iris melanoma [3, 14, 26]. Nonetheless, detection at the earliest stage with smaller tumours limited to the eye and not involving the angle structures would enable early intervention with improved patient survival. Surprisingly, in the current study there was a high prevalence of more advanced clinical features including ectropion uveae, corectopia, cataract, and fewer spindle cell tumours than comparable studies [3, 14]. This may reflect the high UV exposure in NZ and a predominantly white Caucasian population. Notably, the mean five-year melanoma-related metastasis rate was low at 2.0% and similar to international data. Nevertheless, the proportion of high grade tumours (T2-4) in this study (4.1%) was significantly lower than that in the US (25%) [3]. This suggests that the free-to-access public hospital ophthalmology service and widespread community optometry services are capable of recognizing iris tumours early, to allow patients access to high quality care promptly. However, the variable size and extent of iris tumours identified in this study, and the loss of 17.6% of eyes to enucleation, clearly suggests an ongoing need for better public and professional awareness of the need to refer pigmented lesions of the iris at the earliest stage.

### Summary Table

#### What was known before

- Iris melanoma is a rare disease that predominantly occurs in white Caucasian populations.
- Recent evidence suggests ultraviolet radiation exposure is implicated in the development of iris melanoma but there is limited data from high UV-exposure regions such as New Zealand/Aotearoa.
- Management of iris melanomas has evolved with increasing utilisation of new modalities such as proton beam radiotherapy, and decreasing utilization of enucleation.

#### What this study adds

- Iris melanoma was identified in white Caucasians but not in Maori or Pacific peoples in the north island of New Zealand/Aotearoa in 1999-2018.
- Despite the high ultraviolet environment, iris tumour stage appeared less advanced than other international studies, suggesting a relatively well functioning health system with early detection and referral, nonetheless, anterior chamber angle involvement was common.
- Five year outcomes were favourable with only 2% melanoma-related metastasis and death, however, management ultimately included enucleation in 17.6% confirming significant morbidity from iris melanoma even in a relatively wealthy economy with good public health services.

## Data Availability

The datasets analysed during the current study are not publicly available to maintain patient confidentiality and the clear risk of patient identification from this relatively rare condition and small patient population. The datasets are available from the corresponding author on reasonable request.
